# Supplementary material for: Causal Relationship between Aging and Anorexia Nervosa: A White-Matter-Microstructure-Mediated Mendelian Randomization Analysis
Source: Biomedicines. 2024 Aug 16;12(8):1874. doi: 10.3390/biomedicines12081874 (PMC11351342; doi:10.3390/biomedicines12081874)
Supplement: Supplementary file 1 [file biomedicines-12-01874-s001.zip › Supplementary Figure/Figure S4/Singe SNP plot of MR analysis for TL to ubm-b-1812.pdf]

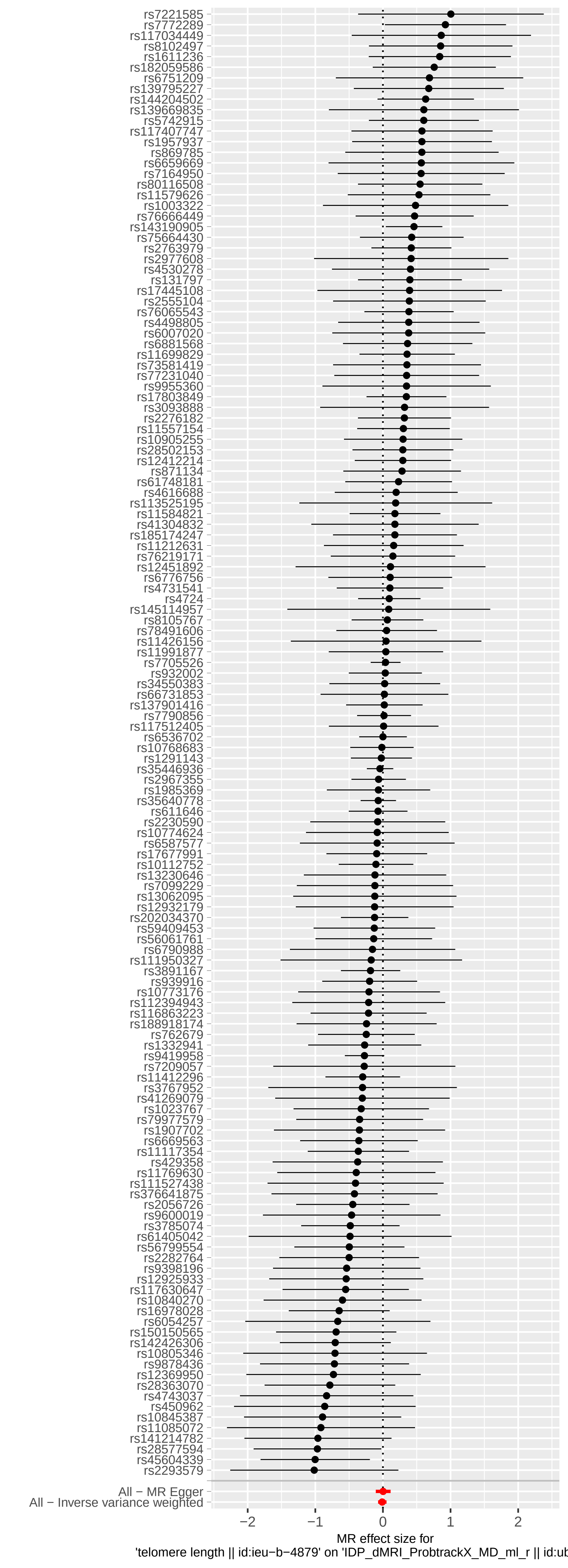
rs7221585
rs7772289
rs117034449
rs8102497
rs1611236
rs182059586
rs6751209
rs139795227
rs144204502
rs139669835
rs5742915
rs117407747
rs1957937
rs869785
rs6659669
rs7164950
rs80116508
rs11579626
rs1003322
rs76666449
rs143190905
rs75664430
rs2763979
rs2977608
rs4530278
rs131797
rs17445108
rs2555104
rs76065543
rs4498805
rs6007020
rs6881568
rs11699829
rs73581419
rs77231040
rs9955360
rs17803849
rs3093888
rs2276182
rs11557154
rs10905255
rs28502153
rs12412214
rs871134
rs61748181
rs4616688
rs113525195
rs11584821
rs41304832
rs185174247
rs11212631
rs76219171
rs12451892
rs6776756
rs4731541
rs4724
rs145114957
rs8105767
rs78491606
rs11426156
rs11991877
rs7705526
rs932002
rs34550383
rs66731853
rs137901416
rs7790856
rs117512405
rs6536702
rs10768683
rs1291143
rs35446936
rs2967355
rs1985369
rs35640778
rs611646
rs2230590
rs10774624
rs6587577
rs17677991
rs10112752
rs13230646
rs7099229
rs13062095
rs12932179
rs202034370
rs59409453
rs56061761
rs6790988
rs111950327
rs3891167
rs939916
rs10773176
rs112394943
rs116863223
rs188918174
rs762679
rs1332941
rs9419958
rs7209057
rs11412296
rs3767952
rs41269079
rs1023767
rs79977579
rs1907702
rs6669563
rs11117354
rs429358
rs11769630
rs111527438
rs376641875
rs2056726
rs9600019
rs3785074
rs61405042
rs56799554
rs2282764
rs9398196
rs12925933
rs117630647
rs10840270
rs16978028
rs6054257
rs150150565
rs142426306
rs10805346
rs9878436
rs12369950
rs28363070
rs4743037
rs450962
rs10845387
rs11085072
rs141214782
rs28577594
rs45604339
rs2293579
All − MR Egger
All − Inverse variance weighted
−2
−1
0
1
2
MR effect size for
'telomere length || id:ieu−b−4879' on 'IDP_dMRI_ProbtrackX_MD_ml_r || id:ub
